# CDK2-AP1 inhibits growth of breast cancer cells by regulating cell cycle and increasing docetaxel sensitivity *in vivo* and *in vitro*

**DOI:** 10.1186/s12935-014-0130-8

**Published:** 2014-11-30

**Authors:** Xiangming He, Hua Xiang, Xiangyun Zong, Xuebing Yan, Yang Yu, Guan Liu, Dehong Zou, Hongjian Yang

**Affiliations:** Department of Breast Surgery, Zhejiang Cancer Hospital, 38 Guangji Road, Hangzhou, 310022 China; Department of Breast Surgery, Shanghai Jiao Tong University affiliated Shanghai sixth Hospital, 600 Yishan Road, Shanghai, 200233 China; Department of Pathology, First Affiliated Hospital of College of Medicine, Zhejiang University, 79 Qingchun Road, Hangzhou, 310003 China; Department of Radiotherapy, Zhejiang Cancer Hospital, 38 Guangji Road, Hangzhou, 310022 China

**Keywords:** CDK2-AP1, Breast cancer, Cell cycle, Chemotherapy sensitivity

## Abstract

**Background:**

Cell cycle regulatory pathway is a well-established pathway mainly dependent on cyclin-dependent kinases (CDKs), which are regulated positively by cyclins and negatively by cyclin-dependent kinase inhibitors(CKIs). Cyclin-dependent kinase 2 associate protein 1(CDK2-AP1) is a specific negative regulatory protein for CDK2, is important in the cancer cell cycle. However, the function of CDK2-AP1 in breast cancer remains unclear. We designed therefore explored the effects of CDK2-AP1 on breast cancer growth and its chemo-sensitivity.

**Methods:**

Expression of CDK2-AP1, CDK2 and CyclinD1 in 209 cases of pathological specimens using IHC staining was measured. Lost-of-function and Gain-of-function assays were used *in vivo* and *in vitro* relating to the specific role of CDK2-AP1 in breast cancer. We analyzed *in vivo* and *in vitro* the impact of CDK2-AP1 on chemotherapy sensitivity in breast cancer.

**Results:**

The positive ratio of CDK2-AP1 expression was reduced successively in normal breast tissue, DCIS, invasive breast cancer and relapsed breast cancer, however, with CDK2 and CyclinD1 it was suggested that CDK2-AP1 was correlated closely with the tumorigenesis and progress, and might work as a tumor suppressor. After down-regulating CDK2-AP1 in breast cancer cells, the cell cycle was accelerated and cell proliferation enhanced. The cell cycle was arrested in G0/G1 phase and G2/M phase after up-regulating CDK2-AP1 in breast cancer cells, inhibiting cell proliferation. The expression of CDK2 and CyclinD1 changed accordingly after downregulation or upregulation of CDK2-AP1 by western blot, suggesting a role of the CDK2-AP1/CDK2/CyclinD1 cell cycle pathway in the initiation and progression of breast cancer. Similar results were obtained in animal assays. The data indicates that CDK2-AP1 can induce sensitivity to docetaxel treatment in breast cancer cells.

**Conclusions:**

CDK2-AP1 affects tumorigenesis, tumor growth and chemo-sensitivity by cell cycle regulation, which can potentially to be a therapeutical agent in breast cancer.

## Background

Despite encouraging advances in clinical and experimental research, breast cancer ranks the first in the morbidity of malignancies in women. Since 2008, the morbidity and mortality of breast cancer have increased by 20 and 14%, respectively. As estimated by World Health Organization (WHO), there were ~1.68 million new cases diagnosed and 522,000 deaths due to breast cancer worldwide in 2012 [1]. Surgery and chemoradiotherapy are conventional treatments, but they are not always satisfactory. Targeting the estrogen/estrogen receptor pathway or the human epidermal growth factor 2 (HER2) pathway now shows promise in therapeutic strategies. However, both have limitations in case selection, which may lead to poor efficacy. Therefore, new targets and related molecular markers need to be identified and urgently investigated.

Cell cycle regulatory pathway is mainly dependent on cyclin-dependent kinases (CDKs), which are regulated positively by cyclins and negatively by cyclin-dependent kinase inhibitors (CKIs). When CDKs are abnormally upregulated, cells will always be undergoing cycles and may acquire resistance to apoptosis, finally resulting in uncontrolled proliferation [2]. CDK2 is a member of CDKs family that is important in malignant transformation of human breast epithelial cells, probably by complexing with cyclinD1 or with the assistance of low molecular weight cyclinE [3-6]. Inhibition of CDK2 activity could effectively restrain the proliferation of breast cancer cells [7,8], including those resistant to endocrinotherapy [9].

Cyclin-dependent kinase 2 associate protein 1(CDK2-AP1) is a specific negative regulatory protein for CDK2, which is mainly responsible for degrading CDK2 and interacting with DNA polymerase α to affect DNA replication of S-phase cells. The CDK-AP1 gene is a highly conserved gene originating from normal keratinized epithelium clones; it is located on chromosome 12q24.31 and has a full length of 1627 bp. It is also known to be deleted in oral cancer-1(DOC-1) or P12, which is mainly expressed in most human tissues, including breast, liver and lung. Todd et al. [10] and Tsuji et al. [11] reported that CDK2-AP1 may be an important tumor suppressor in oral cancer because of its more frequent expression in normal tissues than in oral cancer tissues [10,11]. This speculation was confirmed by studies showing that CDK2-AP1 can control the proliferation of oral cancer cells in a TGF-β-dependent manner [12,13]. Kim et al. [14] have also shown that CDK2-AP1 can promote apoptosis of cancer cells by regulating CDK2 in cases where chemotherapy has caused DNA damage, suggesting a potential therapeutic role combined with DNA-damaging agents (eg cisplatin) for oral/head and neck cancers. In gastric cancer and esophageal cancer, loss of CDK2-AP1 is closely correlated with malignant progression of cancer cells and a poor prognosis for patients [15,16]. CDK2-AP1 also seems to have the potential to control cancer cell growth and modify the functioning of the androgen-responsive pathway, described by Zolochevska et al. [17].

In spite of its important role in cancer suppression, work on CDK2-AP1 in breast cancer is insufficient. Only one single report had indicated that P12 can inhibit growth of breast cancer cells in vivo and in vitro by regulating the cell cycle [18]. However, either its role in cell behavior or chemotherapeutic sensitivity of breast cancer remains unclear. We have followed the expression of CDK2-AP1 and its related molecules (CDK2 and CyclinD1) in normal human breast tissues and breast cancers at different stages. We also did these assays *in vivo* and *in vitro* to explore the specific roles of CDK2-AP1 in breast cancer cells. The findings improve our understanding of the role of CDK2-AP1 in the development of breast cancer and clarify the effect of CDK2-AP1 in chemotherapy of breast cancer.

## Materials and methods

### Tissue specimens

For CDK2-AP1 expression analysis, samples including normal breast tissue, DCIS, invasive breast cancer and relapsed breast cancer from 209 patients were selected randomly from Zhejiang Cancer Hospital from 2008 to 2011. None adjuvant chemoradiotherapies were adopted before surgery. Informed consent for the research was obtained from each patient. The Ethics Committee and the Academic Committee of Zhejiang Cancer Hospital approved this study.

### Immunohistochemical staining

The paraffin blocks were cut into 5 μm sections and mounted on slides. The sections were deparaffinized in xylene and dehydrated with graded ethanol washes. The slides were incubated with rabbit anti-human CDK2-AP1/CDK2/CyclinD1 monoclonal antibody (Cat. #: 2910–1, 1134–1 and 1677–1, Epitomics, Burlingame, CA) overnight at 4°C. The slides were incubated with a biotinylated goat anti-rabbit serum for 30 min and subsequently reacted with a streptavidin-peroxidase conjugate and 3′, 3′-diaminobenzidine. Negative control was prepared using the same procedure, except that normal rabbit IgG was substituted for the primary anti-CDK2-AP1 antibody. The staining intensities were classified into 2 groups: positive (>80% of tumor cells were positively stained), negative (<20%).

### Cell culture

Human breast cancer cell lines MDA-MB-231, SK-BR-3 and MCF-7 were obtained from Chinese Academy of Science Shanghai cell bank (Shanghai). The cells were cultured at 37°C in 5% CO2/95% air in Dulbecco’s Modified Eagle’s Media (DMEM; Lonza) containing 10% FBS (Lonza), 100 units/mL penicillin, and 100 μg/mL streptomycin (Lonza). Parallel cell lines were treated with 50 μg/ml epirubicin or 60 μg/ml docetaxel as contrast test.

### Plasmid construction

The full length cDNA of CDK2-AP1 was amplified from human brain library using the PCR method. CDK2-AP1 cDNA was inserted into the linearized vector pLV3 (Genechem). The products were transformed into bacterial competent cells. Positive colonies with inserted fragments were confirmed by DNA sequencing to generate pLV3-CDK2-AP1 expression plasmid.

 For RNA interference, 21-bp oligonucleotides encoding CDK2-AP1 (NM_004642) -specific siRNA were synthesized. The siRNA sequence targeting CDK2-AP1 was 5′-GCTGCTGGCCATCATTGAAGA -3′, and the non-silencing control was 5′-TTCTCCGAACGTGTCACGT -3′. siRNA oligos were annealed and ligated into the BamH I/EcoR I-linearized pLV3 vector to generate CDK2-AP1 shRNA and control shRNA expressing plasmids.

### Lentivirus packaging

The recombinant lentiviral plasmids, the packaging vector pGag/Pol, pRev, and the envelope vector pVSV-G were co-transfected into HEK293T cells using MISSION Lentiviral Packaging Mix kit (Sigma-aldrich). The mixture, including 20 μL Packing Mix (PVM), 12 μL PEI, and 400 μL serum-free DMEM and 20 μg purified plasmid DNA, was incubated at room temperature for 15 min and transferred to HEK293T cells at 70–80% confluence. The medium was replaced with fresh complete medium after 16 h incubation, and the cells cultured for 48 h. The supernatant were collected, purified, and concentrated with a Centricon Plus-20 centrifugal ultrafiltration device (Millipore).

### Real-time RT-PCR

RNA was isolated using the TRIzol reagent (RNAiso; Invitrogen). RNA was extracted with phenol-chloroform, ethanol precipitated, and resuspended in diethyl pyrocarbonate–treated H2O. cDNA was prepared with a Reverse Transcription kit (Promega) and subjected to quantitative real-time PCR and reverse transcription PCR (RT-PCR).

Primer pairs for CDK2AP1 (GenBank,NM_004642) were 5′-AGCATGGCAACGTC TTCACAGT-3′ and 5′-TGGCATTCCGTTCCGTTTCT-3′. Primer pairs for CDK2 (GenBank, X61622) were 5′-TGGATGCCTCTGCTCTCACT-3′ and 5′-ATATTTCGAGCCCAGGAGGA-3′. Each sample was processed in parallel with assays for GAPDH (5′-ACCACAGTCCATGCCATCAC-3′, 5′-TCCACCACCCTGT TGCTGTA-3′), and the absolute levels of each mRNA were normalized relative to GAPDH.

One microliter template cDNA was used in a real-time qPCR reaction with Kapa Sybr Fast Master Mix (Applied Biosystems) and 10 pM each forward and reverse primers for experimental or control genes. Polymerase was activated at 95°C for 2 min, followed by 40 cycles of denaturation at 95°C for 30 seconds, annealing at 60°C for 30 seconds, and extension at 60°C for 30 seconds, with a final extension at 72°C for 5 min. Reactions were run on a Step One Plus Real-time PCR System (Applied Biosystems).

### Western blot analysis

The protein concentration of the supernatants was determined using the Bio-Rad DC protein assay system, with BSA as standard. Equal amounts of protein (30 μg) were run in 12% SDS-PAGE and transferring onto nitrocellulose membranes (Millipore). Membranes were incubated with primary antibodies, washed, treated with peroxidase-conjugated secondary antibodies, rewashed, and the proteins visualized with enhanced chemiluminescence (ECL; Amersham, UK). Primary antibodies were anti-CDK2-AP1, anti-CDK2, anti-CyclinD1 and anti-GAPDH (BIOSS).

### MTT assay

Cell growth and viability after lentivirus infection for 3 days were measured by the 3-(4,5-dimethylthiazol-2-yl)-2,5-diphenyltetrazolium bromide assay. Cells were plated at 1 × 10^3^/well in 96-well plates, and theirl growth measured after 48 h. Viable adherent cells were stained with 20 μL MTT (5 mg/mL, Sigma-aldrich) for 4 h at 37°C. The medium was removed, and the formazan crystals were dissolved in dimethyl sulfoxide. for measurement of absorbance at 540 nm. Viability was expressed as the ratio relative to untreated cells.

### Colony formation assay

Lentivirus-infected cells in the logarithmic growth phase were digested with trypsin and resuspended in complete medium. One thousand cells/well were plated into 6-well plates in triplicate, which were incubated until most colonies contained >50 cells, with the medium being changed every 3 days. The cells were washed with PBS, and 1 mL paraformaldehyde added to each well for 30 min. They were washed with PBS and stained with 500 μL Giemsa dye for 20 min. The number of colonies in each group was counted.

### PI staining

Cell cycle distribution was analyzed by FACScan flow cytometry (BD Bioscience). MCF-7 cells infected with CDK2-AP1 overexpressing lentivirus were plated at 1 × 10^6^/well in 12-well plates.

After incubation for 24 h, cells were collected, washed with PBS, and fixed with cold 70% ethanol. They were treated with 1 unit DNase-free RNase and incubated for 30 min at 37°C. Propidium iodide (1 mg/mL; Sigma Chemical) was added to the suspension, and a total of 20,000 cells were analyzed by FACScan.

### Tumor formation in nude mice

Cloned pools of CDK2-AP1 interfected MCF-7 cells, CDK2-AP1 overexpressed SK-BR-3 cells and control cells were trypsinized and resuspended in PBS. One million cells/mouse were injected into female athymic nude mice (BALB/c-nu/nu) (n = 54, 20 ± 2 g, 4–5 weeks old, from Shanghai Slac Laboratory Animal Co, Ltd). The mice were treated with saline, epirubicin or docetaxel, 10 mg/kg by tail vein injection each week. The protocol was approved by Ethics and Welfare of Animals committee of Zhejiang Cancer Hospital.

Tumor size was recorded every 3 days with a precision caliper as the maximum diameter (a, mm) and vertical short diameter (b, mm). The volume was calculated using the formula V (mm^3^) = 1/2ab^2^. The mice were killed 5 weeks after tumor cells injection and subcutaneous tumor weight measured. Tumor were detected for bcl2/ bax and CDK2/ cyclinD1 using IHC.

### Statistical analysis

We used SPSS 19.0 software (IBM) for analysis. CDK2-AP1 expression pattern was analyzed using Chi-square test. Tumor weight and velocity was compared by general linear regression for repeated measures. P < 0.05 was considered a significant difference.

## Results

### CDK2-AP1 protein expression in normal and malignant breast tissues and its correlation with CDK2 and CyclinD1

**Figure 1:**
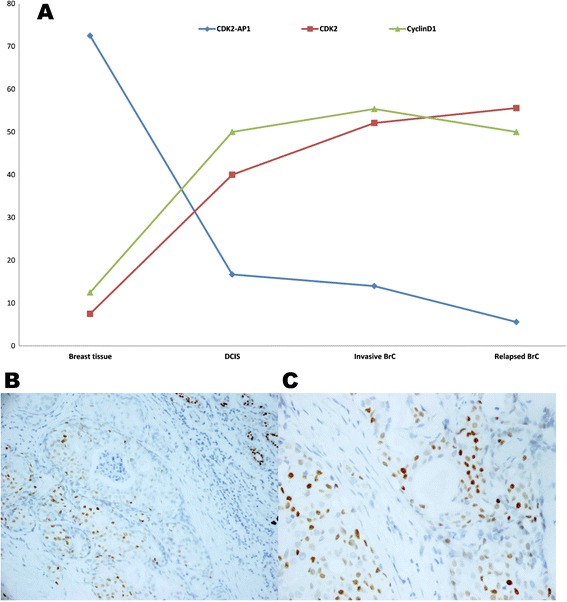
**Expression of CDK2-AP1 is reduced successively in normal breast tissue, DCIS, invasive breast cancer and relapsed breast cancer, contrary to CDK2 and CyclinD1. A**, The positive rates of CDK2-AP1 in normal breast tissue, DCIS, invasive breast cancer and relapsed breast cancer were 72.5, 16.7, 14.0 and 5.6%, respectively. Accordingly the positive rates of CDK2 and CyclinD1 were 7.5, 40.0, 52.1and 55.6%; and 12.5, 50.0, 55.4 and 50%. **B**, Positive staining of CDK2-AP1 (×200) **C**, Positive staining of CDK2-AP1 (×200, in the nucleoli).

Expression of CDK2-AP1, CDK2 and CyclinD1 in 40 cases of normal breast tissues, 30 cases of ductal breast cancer in situ, 121 cases of invasive breast cancer and 18 cases of relapsed breast cancer were examined by immunohistochemical staining. CDK2-AP1 was expressed most frequently in normal breast tissues, and least frequently in metastasized breast cancer. Overall, the positive ratio of expression of CDK2-AP1 was reduced successively in normal breast tissue, DCIS, invasive breast cancer and relapsed breast cancer, in contrast to CDK2 and CyclinD1 expression (Table 1, Figure 1). CDK2-AP1 expression seemed to be correlated closely with the malignant progression, from tumorigenesis to invasion and metastasis. Therefore, CDK2-AP1 could act as a tumor suppressor by inactivating CDK2/CyclinD1, and loss of CDK2-AP1 would be important in the initiation and development of breast cancer.

**Table 1:** **Expression of CDK2-AP1/ CDK2/ CyclinD1 in normal breast tissue, DCIS, invasive breast cancer and relapsed breast cancer**

		**Breast tissue**	**DCIS**	**Invasive BrC**	**Relapsed BrC**	**P**
**CDK2-AP1**	P	29	5	17	1	<0.001
	N	11	25	104	17	
**CDK2**	P	3	12	63	10	<0.001
	N	37	18	59	8	
**CyclinD1**	P	5	15	67	9	<0.001
	N	35	15	54	9	
**Total**		40	30	121	18

### CDK2-AP1 expression knockdown in MCF-7 cells and upregulation in SK-BR-3 cells

**Figure 2:**
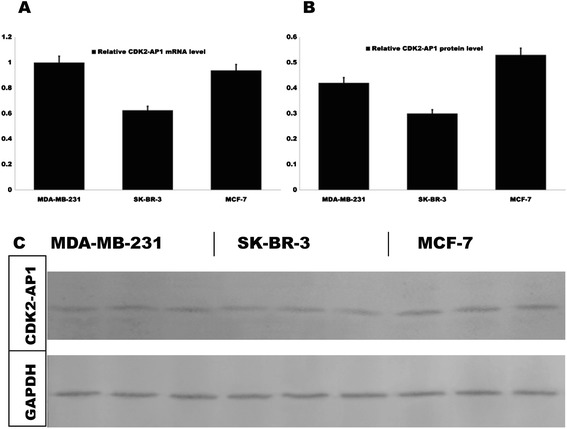
**Expression of CDK2-AP1 mRNA and protein in three breast cancer cell lines.** MCF-7 cells have relatively higher endogenous CDK2-AP1 expression at both mRNA and protein levels. In contrast, SK-BR-3 cells have lower CDK2-AP1 expression. **A**, CDK2-AP1 mRNA level is relatively high in MDA-MB-231 and MCF-7; lower in SK-BR-3. **B**, CDK2-AP1 protein level is higher in MCF-7; lower in SK-BR-3. **C**, Result of western blotting analysis.

**Figure 3:**
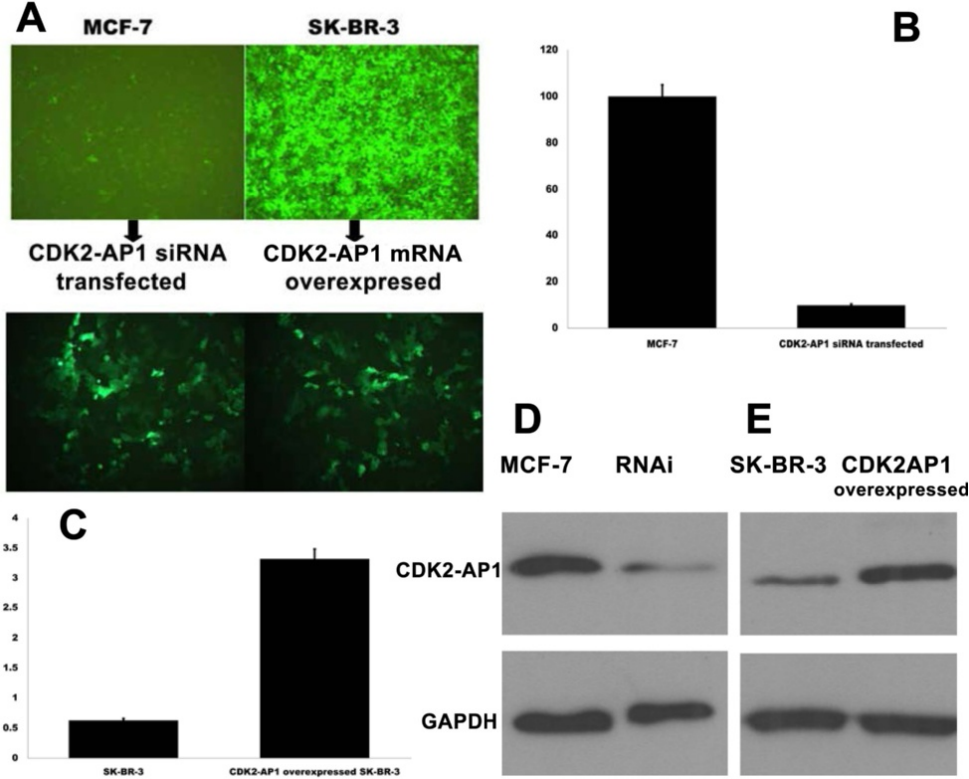
**CDK2-AP1 was knocked down in MCF-7 cells and overexpressed in SK-BR-3 cells. A**, CDK2-AP1 siRNA was transfected into MCF-7 cells and CDK2-AP1 overexpressed lentivirus vector was transfected into SK-BR-3 cells. **B**, CDK2-AP1 RNAi lentivirus infection results in 80.1% reduction of CDK2-AP1 mRNA in RNA interfered MCF-7. **C**, CDK2-AP1 mRNA is upregulated 4.3-fold in CDK2-AP1 overexpressed SK-BR-3 cells compared to the control cells. **D**, CDK2-AP1 protein level is reduced by 77.7% in interfected MCF-7 cells. **E**, CDK2-AP1 protein expression increases 4 fold in overexpressed SK-BR-3.

Expression of CDK2-AP1 mRNA was investigated by real-time PCR analysis in MCF-7, SK-BR-3 and MDA-MB-231 cell lines (Figure 2A). CDK2-AP1 protein was detected by Western blot assay (Figure 2B, 2C). MCF-7 cells have a relatively higher endogenous CDK2-AP1 expression, both in mRNA and protein. In contrast SK-BR-3 cells have lower CDK2-AP1 expression at both mRNA and protein levels. Lentivirus-mediated CDK2-AP1 siRNA was designed to knockdown endogenous CDK2-AP1 expression in MCF-7 cells (Figure 3A), real-time PCR analysis indicated a 80% reduction of its mRNA (Figure 3B). The endogenous CDK2-AP1 protein level was reduced 78% (Figure 3D), and thus CDK2-AP1 RNAi lentivirus significantly suppresses endogenous CDK2-AP1 expression.

A lentiviral expression vector was used to overexpress CDK2-AP1 in SK-BR-3 cells to see its effectiveness as a growth regulator (Figure 3A). CDK2-AP1 mRNA was upregulated 4.3-fold compared to control cells (Figure 3C), consistent with protein expression (Figure 3E).

### CDK2-AP1 as a growth suppressor of breast cancer cells

**Figure 4:**
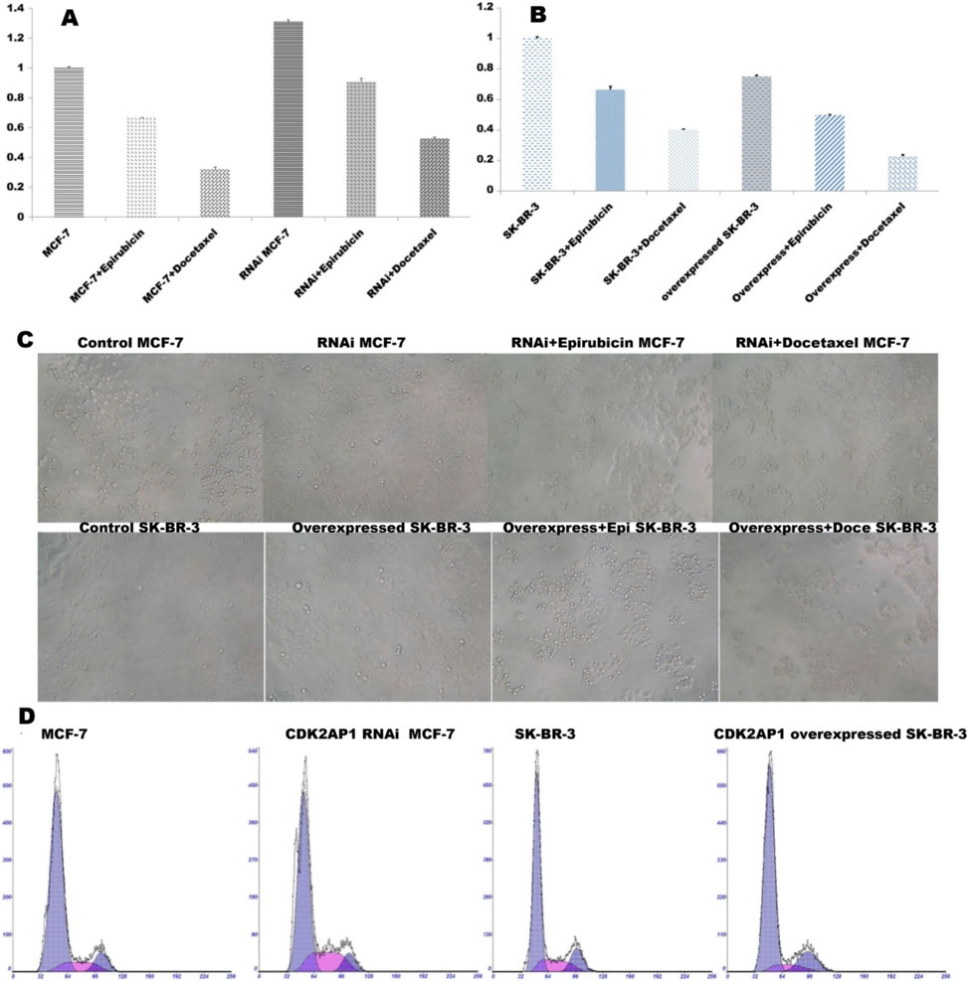
**CDK2-AP1 acts as a growth suppressor of breast cancer cells by arresting cells in G0/G1 and G2/M phase.** Docetaxel sensitivity is increased in breast cancer cells in the case of CDK2-AP1 expression. **A,** after CDK2-AP1 has been knockdown in MCF7 cells, viability was increases by 1.31 fold compared with the control group. **B**, CDK2-AP1 overexpression suppresses the growth of SK-BR-3 cells. **C**, colony number of MCF7 cells transfected with the CDK2-AP1 RNAi lentivirus is twice that of the control group, whereas for SK-BR-3 cells overexpressing CDK2-AP1 it is reduced. **D**, overexpressed CDK2-AP1 arrests SK-BR-3 cells in G0/G1 and G2/M phase, whereas downregulated CDK2-AP1 promotes MCF-7 cells into S phase.

MCF7/control and MCF7/ CDK2-AP1 RNAi cells were seeded at low density (1 × 10^3^/well of 96-well plate), and their growth rates measured by MTT assay. When CDK2- AP1 was knockdown in MCF-7 cells, growth increased by 1.3 fold in comparison with control group 48 h later (Figure 4A). The colony number of MCF7 cells transfected with the CDK2-AP1 RNAi lentivirus was twice as high as the control group (Figure 4C).

 Regarding the effect of CDK2-AP1 overexpression in SK-BR-3 cells, MTT and colony formation assays showed that it suppressed growth and colony formation (Figure 4B,C), both indicators of its growth suppressor activity.

### CDK2-AP1 arrests cells in G0/G1 and G2/M phase

 Overexpressed CDK2-AP1 arrested SK-BR-3 cells in G0/G1 and G2/M phase (Figure 4D). The percentage of G0/G1 phase cells increased from 68 to 77% (P < 0.05), and G2/M phase from 14 to 14.5%, accompanied by a fall of S phase cells from 17.6 to 8.5%. In contrast, in CDK2-AP1 silenced MCF-7 cells G0/G1 and G2/M phase cells decreased from 78.9 to 67.7% and 9.06 to 8.01%, respectively, while S phase cells increased from 12,0 to 24.3%. Thus the CDK2-AP1 level is closely correlated with cell cycle progression.

### Inhibition of CDK2-AP1 in tumorigenesis of MCF-7 cells in nude mice

**Figure 5:**
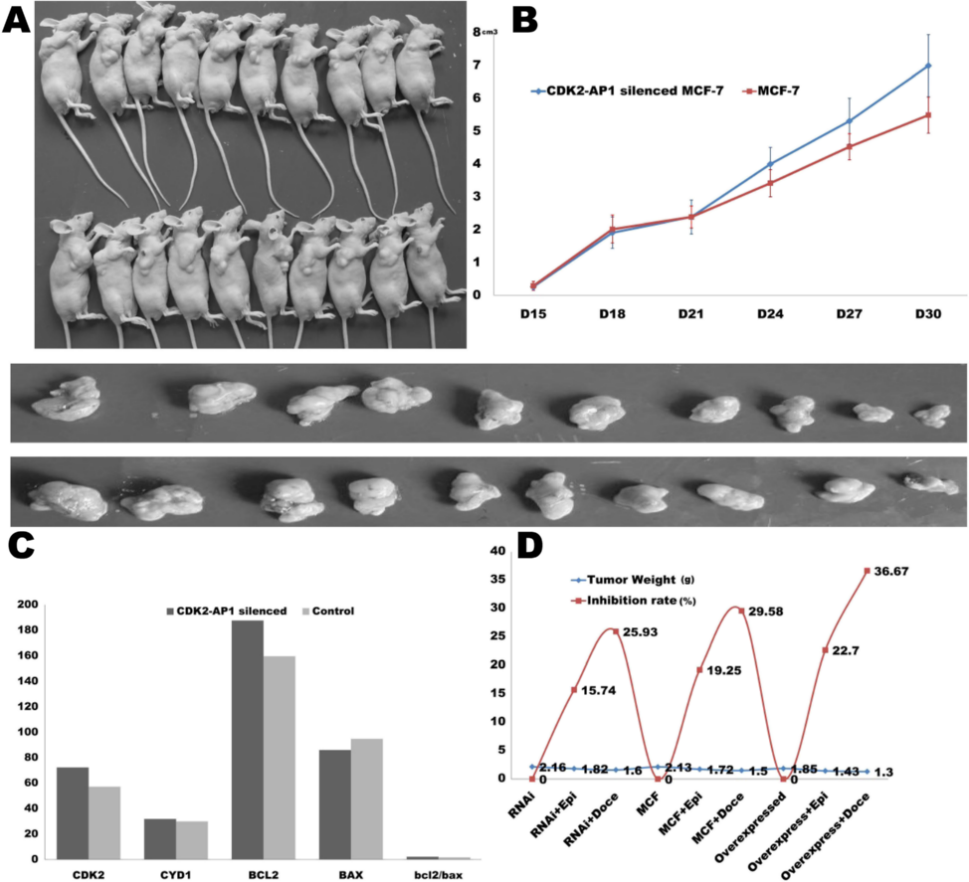
**Loss of CDK2-AP1 promotes tumorigenesis of MCF-7 cells in nude mice, inhibits tumor apoptosis, and lowers sensitivity to docetaxel. A**, inhibition of CDK2-AP1 results in a significant increase in tumor size (p < 0.05). **B**, tumor volumes increase compared to the controls (p < 0.05). **C**, expression of CDK2/ cyclinD1 and the ratio of bcl2/ bax both increase after inhibition of CDK2-AP1 according to the result of IHC. **D**, CDK2-AP1 silenced MCF-7, MCF-7 and CDK2-AP1 overexpressed SK-BR-3 cells have been used in the drug sensitivity experiments, docetaxel sensitivity was increased in the case of CDK2-AP1 expression, whereas silencing CDK2-AP1 induces resistance to treatment in breast cancer (p < 0.05). Expression of CDK2-AP1 had little influence on epirubicin sensitivity.

 To confirm that CDK2-AP1 can be involved in breast cancer development, CDK2-AP1 silenced MCF-7 cells and control cells were injected into nude mice subcutaneously and allowed to grow for 5 weeks. Inhibition of CDK2-AP1 promoted tumor growth with a significant increase in tumor size. Both the tumor volume and weight dramatic ally increased compared to the controls (Figure 5A,B). Immunohistochemistry suggested that CDK2/ cyclinD1 expression and the ratio of bcl2/bax also increased after inhibition of CDK2-AP1 (Figure 5C).

### Silencing CDK2-AP1 and resistance to docetaxel treatment in breast cancer cells in vivo and vitro

 To assess the effect of CDK2-AP1 as a potential chemotherapeutic agent, drug sensitivity assays were run both *in vivo* and *in vitro* (Figures 4A-C and 5D). After treatment with docetaxel, CDK2-AP1-silenced group proved less sensitive to docetaxel treatment, having a higher cell proliferation rate than the controls. After treatment with epirubicin, however, no significant difference between the two groups in terms of cell proliferation were seen, a result confirmed by bearing-tumor nude mice assay. Thus CDK2-AP1 could enhance the sensitivity of docetaxel treatment in breast cancer cells.

## Discussion

CDK2-AP1 is a direct upstream of CDK2, which can induce cell apoptosis by inactivating CDK2. On losing it CDK2-AP1, an activated downstream signal promotes excessive proliferation of normal cells, which may lead to malignant transformation of normal cells. In cancer, however, loss of CDK2-AP1 could increase proliferation and invasiveness to cancer cells. By immunohistochemistry, the expression of CDK2-AP1 was found to be reduced successively in normal breast tissue, DCIS, invasive breast cancer and metastasized breast cancer. The expression of CDK2-AP1 was also negatively correlated with the expression of CDK2 and cyclin D1. We deduced from this that loss of CDK2-AP1 is important in the development of breast cancer; loss of CDK2-AP1 might promote the progression of breast cancer through altered downstream cell cycle-related molecules.

Loss-of-function and gain-of-function assays helped assess the specific role of CDK2-AP1 in breast cancer. After downregulating CDK2-AP1 in breast cancer cells, cell proliferation rate increased, whereas after upregulating CDK2-AP1, there was arrest in G0/G1 phase and G2/M phase and therefore inhibited proliferation. To find whether these changes were due to the regulation of CDK2-AP1 in CDK2-AP1/CDK2/CyclinD1 pathway, we looked at the expression of CDK2 and CyclinD1. Both were changed accordingly after downregulating or upregulating CDK2-AP1, which indicates a possible role of the CDK2-AP1/CDK2/CyclinD1 cell cycle pathway in the initiation and progression of breast cancer. This was confirmed by animal assays which showed that: 1) a CDK2-AP1 silenced group was more tumorigenic and had a faster growth rate of tumor than the controls; 2) higher expression of CDK2 and CyclinD1 was seen, accompanied by a higher ratio of bcl2/bax in the CDK2-AP1 silenced group than in the control group. These results imply the following: loss of CDK2-AP1 activates CDK2 and CyclinD1 continuously, which in turn increases the ratio of bcl2/bax probably by upregulating bcl2, which finally enhances resistance to apoptosis in the cancer cells. In conclusion, the findings offer evidence that loss of CDK2-AP1 promotes the initiation and progression of breast cancer by its effects on the cell cycle.

 We also assayed *in vivo* and *in vitro* the impact of CDK2-AP1 on chemotherapeutic sensitivity in breast cancer. After treating cancer cells with docetaxel, the CDK2-AP1- silenced group had a faster rate of cell proliferation and tumor growth than the control group, suggesting that CDK2-AP1 enhances the sensitivity. However, cancer cells treated with epirubicin showed no such difference. We surmise that epirubicin works as a non-specific cell-cycle agent which could disturb transcription and inhibit the synthesis of DNA and RNA by being directly inserted into DNA base-pairs. Thus, it is to be expected that a CDK2-AP1 activated cell cycle pathway has little effect on epirubicin treatment. Notably, the anti-tumor effect of docetaxel is mainly based on its suppression of cell division and proliferation by inducing tubulin polymerization, which prevents spindle formation during mitosis. Therefore, by its differential effects on the phases of cell cycle, CDK2-AP1 induces sensitivity to docetaxel treatment in breast cancer cells.

In summary, abnormal CDK2-AP1 expression is associated with the development and progression of breast cancer. It also acts on the sensitivity to docetaxel in breast cancer. Although the functions of CDK2-AP1 in cancer are not yet fully understood, these new insight into CDK2-AP1 in the biology and treatment of breast cancer should help. CDK2-AP1 has potential value as a new agent for the prevention and therapy of breast cancer.
